# Muscle Fiber Type-Dependent Differences in the Regulation of Protein Synthesis

**DOI:** 10.1371/journal.pone.0037890

**Published:** 2012-05-22

**Authors:** Craig A. Goodman, Jack A. Kotecki, Brittany L. Jacobs, Troy A. Hornberger

**Affiliations:** Department of Comparative Biosciences, School of Veterinary Medicine, University of Wisconsin-Madison, Madison, Wisconsin, United States of America; University of Padova, Italy

## Abstract

This study examined fiber type-dependent differences in the regulation of protein synthesis in individual muscle fibers found within the same whole muscle. Specifically, the *in vivo* SUrface SEnsing of Translation (SUnSET) methodology was used to measure protein synthesis in type 1, 2A, 2X and 2B fibers of the mouse plantaris muscle, in response to food deprivation (FD), and mechanical overload induced by synergist ablation (SA). The results show that 48 h of FD induced a greater decrease in protein synthesis in type 2X and 2B fibers compared to type 1 and 2A fibers. Type 2X and 2B fibers also had the largest FD-induced decrease in total S6 protein and Ser^240/244^ S6 phosphorylation, respectively. Moreover, only type 2X and 2B fibers displayed a FD-induced decrease in cross-sectional area (CSA). Ten days of SA also induced fiber type-dependent responses, with type 2B fibers having the smallest SA-induced increases in protein synthesis, CSA and Ser^240/244^ S6 phosphorylation, but the largest increase in total S6 protein. Embryonic myosin heavy chain (MHC^Emb^) positive fibers were also found in SA muscles and the protein synthesis rates, levels of S6 Ser^240/244^ phosphorylation, and total S6 protein content, were 3.6-, 6.1- and 2.9-fold greater than that found in fibers from control muscles, respectively. Overall, these results reveal differential responses in the regulation of protein synthesis and fiber size between fiber types found within the same whole muscle. Moreover, these findings demonstrate that changes found at the whole muscle level do not necessarily reflect changes in individual fiber types.

## Introduction

Mammalian skeletal muscles are heterogeneous tissues composed of different fiber types that are identified by their expression of specific myosin heavy chain (MHC) isoforms. For example, adult limb and trunk muscles are composed of four major fiber types; oxidative slow-twitch type 1 fibers, oxidative fast-twitch type 2A fibers, and glycolytic fast-twitch type 2X and 2B fibers, which express MHC isoforms 1, 2a, 2x and 2b, respectively. Over the last century, a large body of research has described the distinct genetic, structural, functional, metabolic and adaptive characteristics of these fiber types (for reviews see [1]–[5]). However, due to technical limitations, comparatively little is known about possible fiber type-dependent differences in the regulation of protein synthesis [6]. Nonetheless, a thorough understanding of how protein synthesis is regulated in different fiber types remains fundamentally important to furthering our knowledge of how skeletal muscles adapt to conditions such as altered mechanical loading, injury, changes in nutritional or hormonal status, and to pharmacological or genetic therapies [5].

Recently, we developed a non-radioactive method for measuring *in vivo* rates of protein synthesis at the single muscle fiber level. This method is based on the principles of the SUrface SEnsing of Translation (SUnSET) technique and it involves injecting animals with a low dose of puromycin (a structural analog of tyrosyl-tRNA) [7], [8]. The puromycin gets incorporated into nascent peptides in a rate-dependent manner and then anti-puromycin immunohistochemistry is used to quantify the amount of puromycin labelled peptides [8]. Importantly, several studies have shown that the quantitative results obtained with this approach are indistinguishable from those obtained with more traditional methods of measuring protein synthesis [7]–[9]. Furthermore, when combined with immunohistochemistry against specific MHC isoforms, this methodology can be used to determine rates of protein synthesis within a specific muscle fiber type [8]. Indeed, we have successfully used the SUnSET approach to demonstrate that basal rates of protein synthesis in the mouse plantaris (PLT) muscle vary in a fiber-type dependent manner (2B < 2X < 2A ≈ 1) [8].

Our finding of fiber type-dependent differences in basal rates of protein synthesis highlights the long standing question of whether physiological perturbations can alter protein synthesis in a fiber type-dependent manner [10]. In support of this possibility, it has been shown that perturbations, such as food deprivation, alcohol administration, glucocorticoids, and burns, induce a greater decrease in the rate of protein synthesis in whole muscles that are composed predominantly of fast-twitch fibers (e.g. extensor digitorum longus vs. soleus) [11]–[15]. These whole muscle studies seemingly provide support for the hypothesis that the adaptive changes in protein synthesis occur in a fiber type-dependent manner. However, it could also be argued that the differences observed between whole fast- and slow-twitch muscles have nothing to do with fiber type composition per se, but instead, are the result of their different anatomical positions and functional roles. Hence, we set out to firmly establish whether or not various stimuli can induce fiber type specific alterations in the rate of protein synthesis within a single whole muscle. To accomplish this goal, we subjected mice to food deprivation, or mechanical overload via synergist ablation (SA), and then used the SUnSET approach to measure the effect on protein synthesis in type 1, 2A, 2X and 2B fibers of the PLT muscle. Based on our results, it is now apparent that physiological perturbations can induce fiber type specific changes in the rate of protein synthesis.

## Materials and Methods

### Animals

Male FVB/N mice, 8–10 wk old, were used for all conditions. Mice were housed under a 12 h light/dark cycle with *ad libitum* access to food and water unless otherwise stated. Before all surgical procedures, mice were anesthetized with an intraperitoneal (IP) injection of ketamine (100 mg/kg) and xylazine (10 mg/kg). After tissue extractions, the mice were euthanized by cervical dislocation. All methods were approved by the Institutional Animal Care and Use Committee of the University of Wisconsin-Madison under the protocol #V01324.

### Experimental Models

#### Food Deprivation

Food deprived (FD) mice had food withdrawn for 48 h, with ad libitum access to water as previously described [8]. Control mice were maintained on the ad libitum diet (Ad Lib). After 48 h, the PLT muscles from these mice were subjected to the various measurements described below.

#### Synergist Ablation of the Plantaris Muscle

Mechanical overload was induced by synergist ablation (SA) surgery that involved the bilateral removal of the soleus and distal half of the gastrocnemius muscle, leaving the PLT as the sole plantar flexor muscle [8], [16]. Mice in the control group received a sham surgery, for which an incision was made on the lower leg and then closed. After the surgical procedures, the incision was closed with Vetbond surgical glue (Henry Schein, Melville, NY, USA). Mice were allowed to recover for 10 d, after which, their PLT muscles were subjected to the various measurements described below.

### Measurement of *In Vivo* Protein Synthesis with SUnSET

For *in vivo* measurements of protein synthesis, a pair of mice (Ad lib and FD or Sham and SA) were anesthetized and then simultaneously given an IP injection of 0.04 μmol/g puromycin dissolved in 100 μl of phosphate buffered saline (PBS), as previously described [8]. At exactly 30 min after the injection, the PLT muscles from both mice were simultaneously dissected, aligned adjacent each other in optimal cutting temperature (OCT) compound (Tissue-Tek; Sakura, Torrance, CA, USA) and then frozen together in liquid N_2_-chilled isopentane.

### Immunohistochemical Analysis

#### Muscle Fiber Type, Cross-Sectional Area and Protein Synthesis

Cross sections (10 μm thick) from the paired muscles were taken at the mid-belly and fixed in −20°C acetone for 10 min. Sections were warmed to room temperature for 5 min and then incubated in PBS for 15 min, followed by a 1 h incubation in solution A [PBS with 0.5% bovine serum albumin (BSA) and 0.5% Triton X-100] containing anti-mouse IgG Fab (1∶10; Jackson Immuno-Research). After three 5 min washes with PBS, samples were incubated for 1 h with solution A containing primary antibodies [mouse IgG2a monoclonal anti-puromycin (clone 12D10, 1∶1000) [7] and one, or two, of the following: mouse IgG2b monoclonal anti-type 1 MHC (clone BA-D5, 1∶100), mouse IgG1 monoclonal anti-type 2a MHC (clone SC-71, 1∶100), mouse IgM monoclonal anti-type 2b MHC (clone BF-F3, 1∶10), mouse IgM monoclonal anti-type 2x MHC (clone 6H1, 1∶10), or mouse IgG1 monoclonal anti-embryonic MHC (clone F1.652, 1∶50)]. All MHC antibodies were obtained from the Developmental Studies Hybridoma Bank at the University of Iowa (Ames, IA, USA). After three 5 min washes with PBS, samples were incubated for 1 h with solution A containing secondary antibodies [DyLight 594-conjugated anti-mouse IgG Fc 2a (1∶500) and, depending on the anti-MHC primary antibody applied, FITC-conjugated anti-mouse IgG Fc 1 (1∶100; Jackson Immuno-Research), Alexa 350-conjugated anti-mouse IgG Fc 2b (1∶500; Invitrogen, Carlsbad, CA, USA) or AMCA-conjugated anti-mouse IgM (1∶150; Jackson ImmunoResearch)]. Samples were then washed 3 times for 10 min and images of the different fluorophores were captured with a Nikon DS-QiMc camera (Nikon, Tokyo, Japan), which has a >33-fold working linear range (*r*  =  0.9997, data not shown). The camera was mounted on a Nikon 80i epifluorescence microscope, and monochrome images were captured through TRITC, FITC and/or DAPI cubes and merged with Nikon NIS-Elements D software as previously described [8], [17]. For most of the quantification procedures, up to sixty fibers, within a given fiber type (type 1, 2A, 2X, or 2B), were randomly selected from each control (Ad lib or Sham) and experimental (FD or SA) sample on the same slide. The periphery of these fibers was traced with Nikon NIS-Elements D software and measurements of the area and the average puromycin signal intensity were obtained. To calculate relative rates of protein synthesis, the intensity of the puromycin signal in a given fiber type was expressed relative to the mean intensity obtained from the same fiber type within the control section on the same slide. One exception to this procedure involved the analysis of fibers in SA muscles that expressed the embryonic MHC isoform (MHC^Emb^). For MHC^Emb^ fibers, the area and average puromycin signal intensity was expressed relative to the mean of sixty randomly selected, non-typed, fibers from the sham section on the same slide. All analyses were performed on images that had signal intensities within the linear range of the camera. The analyses were performed by investigators masked to the sample identity and involved the quantification of at least five independent pairs of muscles.

#### Muscle Fiber Type, Total Ribosomal S6 Protein and S6 phosphorylation

Muscle samples were collected, sectioned and stained as described above with the following modifications. After warming and incubation in PBS for 15 min, the sections were then incubated in solution B [PBS with 5% normal goat serum (Jackson Immuno-Research) and 0.5% Triton-X] for 1 h. After three 5 min washes, samples were incubated for 1 h with solution A containing primary antibodies specific for MHC 1, 2a, 2x or 2b as described above, and either rabbit anti-ribosomal S6 protein antibody (1∶100, clone 5G10, Cell Signaling) or anti-rabbit phospho-S6 ribosomal protein (Ser240/244) XP^®^ antibody (1∶100, clone D68F8, Cell Signaling). After three 5 min washes with PBS, samples were incubated for 1 h with solution B containing secondary antibodies for the detection of specific MHC isoforms and DyLight 594-conjugated goat anti-rabbit IgG (1∶8000 for total S6 and 1∶3000 for Ser^240/244^ S6 phosphorylation; Jackson Immuno-Research). Samples were then washed 3 times for 10 min with PBS and images of the different fluorophores were then captured, processed and quantified for relative levels of total S6 and Ser^240/244^ S6 phosphorylation as described for the protein synthesis measurements. One exception to this procedure involved the analysis of basal levels total S6 and Ser^240/244^ S6 phosphorylation in fibers from Ad Lib muscles (see Fig. S3). For these analyses, up to sixty type 2A, and either type 1, 2X, or 2B fibers, were randomly selected from each Ad Lib section, and the average total S6 or Ser^240/244^ S6 phosphorylation signal intensity in individual type 1, 2X, or 2B fibers was expressed relative to the mean total S6, or Ser^240/244^ S6 phosphorylation signal in type 2A fibers from the same section [8].

### Statistical Analysis

All statistical analyses were performed with GraphPad Prism 5.0 (GraphPad Software Inc., La Jolla, CA, USA). Student's 2-tailed, unpaired *t-*tests, were used for all 2-group comparisons. One-way ANOVA with Newman-Keul post-hoc analysis was used for comparisons involving more than 2 groups. The statistical significance level was set at P<0.05.

## Results

### Muscle Fiber Protein Synthesis and Cross Sectional Area

To explore potential fiber type differences in the regulation of protein synthesis, we employed two experimental models that are known to induce a decrease [food deprivation (FD)] and an increase [synergist ablation (SA)] in skeletal muscle protein synthesis [8], [11], [18].

#### Food Deprivation

**Figure 1:**
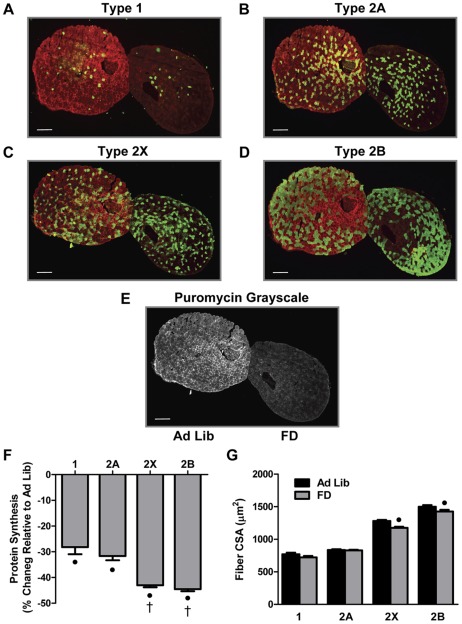
Food Deprivation Induces Fiber Type-Dependent Changes in Protein Synthesis and Cross-Sectional Area. Plantaris muscles obtained from control (Ad Lib) and 48 h food deprived (FD) mice were frozen adjacent to one another, cross-sectioned, and then subjected to immunohistochemistry for rates of protein synthesis (puromycin, red) and fiber type via the identification of (**A**) type 1, (**B**) type 2a, (**C**) type 2x or (**D**) type 2b, myosin heavy chain isoforms (green). (**E**) Grayscale image of the puromycin signal from the same pair of muscles shown in *A–D*. (**F**) The effect of FD on the relative rate of protein synthesis and (**G**) cross-sectional area (CSA), within each fiber type. The bars in A–E indicate a length of 200 μm. All values are presented as the mean + SEM (n  =  60–250 fibers / group from 5 independent pairs of muscles). • Significant effect of FD within a given fiber type, † significantly different from type 1 and 2A fibers, (P<0.05).

In the PLT muscle we identified four different fiber types: slow-twitch type 1 and fast-twitch types 2A, 2X and 2B (Fig. 1 A–D). As shown in Fig. 1E, whole muscle cross-sections of Ad Lib and FD muscles demonstrate that FD resulted in substantial decrease in the overall puromycin signal intensity / protein synthesis. When quantified in the individual fiber types, a significant reduction in protein synthesis in all four fiber types was observed (Fig. 1F and Fig. S1). Furthermore, in type 2X and 2B fibers, the magnitude of the reduction in protein synthesis was significantly greater than that observed in type 1 and 2A fibers (Fig. 1F). Consistent with the larger decreases in protein synthesis, it was also determined that FD only induced a significant decrease in the cross-sectional area (CSA) of the type 2X and 2B fibers (Fig. 1G and Fig. S1). Combined, these data demonstrate that the magnitude of the effect of FD on both protein synthesis, and fiber size, occurs in a fiber-type dependent manner.

#### Synergist Ablation

**Figure 2:**
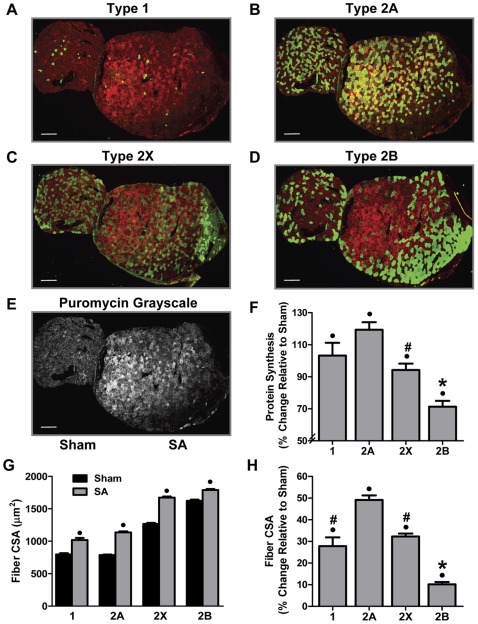
Synergist Ablation Induces Fiber Type-Dependent Changes in Protein Synthesis and Cross-Sectional Area. Plantaris muscles obtained from control (Sham) and 10 d synergist ablated (SA) mice were frozen adjacent to one another, cross-sectioned, and then subjected to immunohistochemistry for rates of protein synthesis (puromycin, red) and fiber type via the identification of (**A**) type 1, (**B**) type 2a, (**C**) type 2x or (**D**) type 2b, myosin heavy chain isoforms (green). (**E**) Grayscale image of the puromycin signal from the same pair of muscles shown in *A–D*. (**F**) The effect of SA on the relative rate of protein synthesis within each fiber type. (**G**) The absolute and (**H**) relative effect of SA on the cross-sectional area (CSA) of each fiber type. The bars in A–E indicate a length of 200 μm. All values are presented as the mean + SEM (n  = 84–500 fibers / group from 6 independent pairs of muscles). • Significant effect of SA within a given fiber type, * significantly different from type 1, 2A and 2X fibers, **#** significantly different from type 2A fibers, (P<0.05).

As shown in Fig. 2A–E, 10 d of synergist ablation (SA) induced a striking hypertrophic response. The hypertrophic effect of SA was also associated with a marked increase in protein synthesis at the whole muscle level (Fig. 2E). When quantified in the individual fiber types, a significant increase in protein synthesis in all four fiber types was observed (Fig. 2F and Fig. S2). These results also demonstrated that type 2B fibers had a significantly smaller increase in protein synthesis when compared to all of the other fiber types (Fig. 2F). Furthermore, the increase in the rate of protein synthesis in type 2X fibers was significantly greater than type 2B fibers but less than type 2A fibers, while the increase in type 1 fibers was not different from that of type 2A and 2X fibers (Fig. 2F). SA also induced a significant increase in the CSA of all four fiber types (Fig. 2G and Fig. S2). Consistent with the changes in protein synthesis, the type 2B fibers also revealed the smallest increase in CSA, while type 2A fibers had the largest increase in CSA (Fig. 2H). Furthermore, the increase in the CSA of type 1 and 2X fibers was greater than that in type 2B fibers, but less than that in type 2A fibers (Fig. 2H). Taken together, these data firmly demonstrate that SA induces muscle fiber type-dependent increases in both protein synthesis and fiber CSA.

### Total Ribosomal S6 Protein and S6 Ser^240/244^ Phosphorylation

To gain insight into the mechanism(s) responsible for the observed fiber type-dependent changes in protein synthesis we performed IHC analysis for crude markers of translational capacity and translational efficiency. As a marker for translational capacity we measured the total amount of the ribosomal S6 protein (a protein associated with the 40S ribosomal subunit [19]). For translational efficiency, we chose to examine changes in Ser^240/244^ phosphorylation of the ribosomal S6 protein. This selection was based on previous studies which have shown that ribosomal S6 protein is phosphorylated at the Serine 240 and 244 residues by p70 ribosomal S6 kinase (p70^S6K^) [19]–[21]. Furthermore, p70^S6K^ is a direct downstream target of the mammalian target of rapamycin complex 1 (mTORC1), and mTORC1 has been widely implicated in the regulation of translational efficiency [22]. Thus, changes in S6 Ser^240/244^ phosphorylation were used as a marker of potential mTORC1-mediated changes in translational efficiency.

#### Food Deprivation

**Figure 3:**
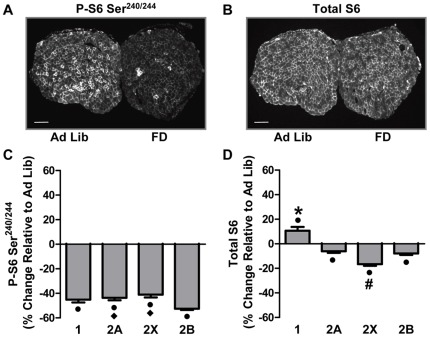
Food Deprivation Induces Fiber Type-Dependent Changes in Ser^240/244^ Phosphorylated and Total Ribosomal S6 Protein. Plantaris muscles obtained from control (Ad Lib) and 48 h food deprived (FD) mice were frozen adjacent to one another, cross-sectioned, and then subjected to immunohistochemistry for different fiber types as described in Figure 1, and Ser^240/244^ phosphorylated S6 (P-S6 Ser^240/244^) or total S6. (**A**) Representative images of P-S6 Ser^240/244^ and (**B**) total S6. (**C**) The effect of FD on the relative amount of P-S6 Ser^240/244^ and (**D**) total S6, within each fiber type. The bars in A and B indicate a length of 200 μm. All values are presented as the mean + SEM (n  = 60–250 fibers / group from 5 independent pairs of muscles). • Significant effect of FD within a given fiber type, **♦** significantly different from type 2B fibers, * significantly different from type 2A, 2X and 2B fibers, **#** significantly different from type 1, 2A and 2B fibers, (P<0.05).

Figures 3A and 3B show Ad Lib and FD muscle cross-sections stained for Ser^240/244^ S6 phosphorylation and total S6 protein, respectively. The first striking observation from these images was the inter-fiber variability of staining intensities within the Ad Lib sections, suggesting the possibility of fiber type-dependent differences in basal levels of Ser^240/244^ S6 phosphorylation and total S6 protein. Indeed, when quantified at the single fiber level, Ser^240/244^ S6 phosphorylation in each fiber type varied in the following manner: type 1 > 2A > 2X  =  2B (Fig. S3A). Furthermore, total S6 protein also varied in a fiber type-dependent manner with type 2A > 1 > 2X > 2B (Fig. S3B). The second striking observation from Fig. 3 was the large FD-induced decrease in Ser^240/244^ S6 phosphorylation (Fig. 3A) and the comparatively small decrease in whole muscle total S6 staining intensity (Fig. 3B). This suggests that FD induced a large decrease in mTORC1 signaling, and that the FD-induced decrease in protein synthesis was more likely due to an acute decrease in translational efficiency rather than a marked decrease in translational capacity. Similar to that observed for the whole muscle sections, Ser^240/244^ S6 phosphorylation was significantly decreased in all four fiber types (Fig. 3C and Fig. S4). The change in Ser^240/244^ S6 phosphorylation also revealed a fiber type-dependent effect with type 2B fibers having a significantly larger decrease than type 2A and 2X fibers (Fig. 3C). Also, relatively small, but significant, fiber type-dependent changes in total S6 protein were observed with total S6 slightly increasing in type 1 fibers and decreasing in 2A, 2X and 2B fibers (Fig. 3D and Fig. S4). Interestingly, the FD-induced decrease in total S6 protein was significantly greater (>2-fold) in type 2X fibers compared to type 2A and 2B fibers. Overall, type 2X and 2B fibers, which had the largest FD-induced decreases in protein synthesis (Fig. 1F), and were the only fiber types to display a FD-induced decrease in CSA (Fig. 1G), also had the largest decreases in total S6 and Ser^240/244^ S6 phosphorylation, respectively. These results suggest that the FD-induced fiber type-dependent changes in protein synthesis are associated with a complex combination of fiber type-dependent changes in translational efficiency, and to a lesser extent, translational capacity.

#### Synergist Ablation

**Figure 4:**
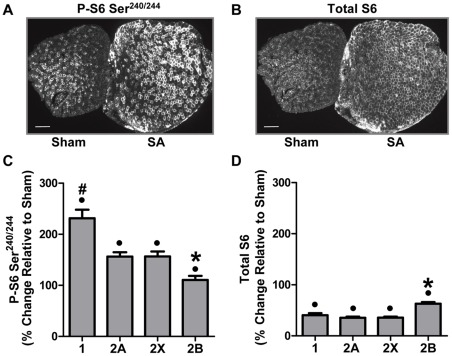
Synergist Ablation Induces Fiber Type-Dependent Changes in Ser^240/244^ Phosphorylated and Total Ribosomal S6 Protein. Plantaris muscles obtained from control (Sham) and 10 d synergist ablated (SA) mice were frozen adjacent to one another, cross-sectioned, and then subjected to immunohistochemistry for different fiber types as described in Figure 2, and Ser^240/244^ phosphorylated S6 (P-S6 Ser^240/244^) or total S6. (**A**) Representative images of P-S6 Ser^240/244^ and (**B**) total S6. (**C**) The effect of SA on the relative amount of P-S6 Ser^240/244^ and (**D**) total S6, within each fiber type. The bars in A and B indicate a length of 200 μm. All values are presented as the mean + SEM (n  = 84–500 fibers / group from 6 independent pairs of muscles). • Significant effect of SA within a given fiber type, ∗ significantly different from type 1, 2A and 2X fibers, **#** significantly different from type 2A and 2X fibers, (P<0.05).

In contrast to FD, SA induced large increases in both Ser^240/244^ S6 phosphorylation (Fig. 4A) and total S6 protein (Fig. 4B) at the whole muscle level. Together, these data suggest that the SA-induced increase in protein synthesis (Fig. 2) was due to a combination of increases in both translational efficiency and translational capacity. At the single fiber level, all fiber types showed a significant increase in Ser^240/244^ S6 phosphorylation; however, this occurred in a fiber type-dependent manner with type 1 fibers revealing the largest increase and type 2B fibers having the smallest increase (Fig. 4C and Fig. S5). Total S6 protein also increased across all the fiber types, and again, the increase was fiber type-dependent, with type 2B fibers having a larger increase in total S6 protein than all other fiber types (Fig. 4D and Fig. S5). Thus, type 2B fibers had the smallest increase in protein synthesis and Ser^240/244^ S6 phosphorylation but the largest increase in total S6 protein. These results highlight that the fiber type-dependent regulation of protein synthesis in response to SA involves a complex interplay between fiber type-dependent changes in translational efficiency and translational capacity.

### MHC^Emb^ Positive Fibers in Synergist Ablated Muscles

Recently, we reported that SA induces a significant increase in the total number of muscle fibers per cross-section [16]. Furthermore, we also showed that SA induced an increase in the number of muscle fibers expressing the embryonic MHC isoform (MHC^Emb^; the isoform predominantly expressed in newly formed fibers), and that the magnitude of the increase in these fibers was similar to the increase in the total number of fibers [16]. Taken together, these data suggested that SA induces the formation of new muscle fibers (i.e. hyperplasia). In the current study, we observed a population of smaller diameter fibers with very high puromycin staining and hypothesized that these might be newly formed MHC^Emb^ positive fibers. Therefore, we stained for the presence of fibers expressing MHC^Emb^ and measured protein synthesis, Ser^240/244^ S6 phosphorylation and total S6 protein in these fibers when compared to a population of mixed fiber types randomly chosen from the adjacent Sham cross-section (Mixed Sham).

**Figure 5:**
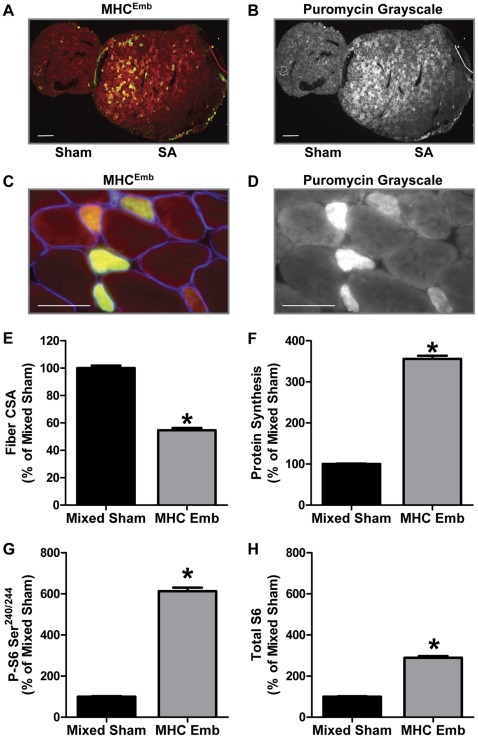
Cross-Sectional Area, Protein Synthesis, Ser^240/244^ Phosphorylated and Total Ribosomal S6 Protein in MHC^Emb^ Positive Fibers. Plantaris muscles obtained from control (Sham) and 10 d synergist ablated (SA) mice were frozen adjacent to one another, cross-sectioned, and then subjected to immunohistochemistry for MHC^Emb^ and rates of protein synthesis (puromycin), Ser^240/244^ phosphorylated S6 (P-S6 Ser^240/244^), or total S6, as described in Figures 2 and 4. (A) Representative image of the signals for puromycin (red) and MHC^Emb^ (green). (B) Grayscale image of the puromycin signal shown in *A*. (C) Higher magnification image from a SA muscle that was tripled stained for puromycin (red), MHC^Emb^ (green) and laminin (blue). (D) Grayscale image of the puromycin signal shown in *C.* (E) The relative cross-sectional area (CSA), (F) rate of protein synthesis, (G) amount of P-S6 Ser^240/244^ and (H) total amount of S6 in MHC^Emb^ positive fibers of SA muscles expressed relative to randomly selected fibers from sham muscles (Mixed Sham). The bars in A and B indicate a length of 200 μm, and the bars in C and D indicate 50 μm in length. All values are presented as the mean + SEM (n  = 152–360 fibers / group from 6 independent pairs of muscles). ∗ Significantly different from mixed sham, (P<0.05).

As shown in Fig. 5A, and as previously reported [16], SA muscles contained a population of MHC^Emb^ positive fibers that were not present in Sham cross-sections. Consistent with the idea that these are newly formed fibers, the MHC^Emb^ positive fibers were significantly smaller in CSA when compared to the Mixed Sham fibers (Fig. 5E and Fig. S6A). Furthermore, higher magnification images revealed that these smaller MHC^Emb^ positive muscle fibers had very high puromycin signal intensities, and thus, high rates of protein synthesis (Fig. 5C and 5D). Indeed, when quantified at the single fiber level, the rate of protein synthesis in MHC^Emb^ positive muscle fibers was 3.6-fold greater than that observed in the Mixed Sham fibers (Fig. 5F and Fig. S6B). Moreover, the higher rates of protein synthesis in the MHC^Emb^ positive fibers was associated with a 6.1-fold greater amount of Ser^240/244^ S6 phosphorylation (Fig. 5G and Fig. S6C) and 2.9-fold greater amount of total S6 (Fig. 5H and Fig. S6D). These results indicate that the putative newly formed MHC^Emb^ positive fibers are rapidly adapting to SA, with increases in mTORC1 signaling, translational efficiency, translational capacity and protein synthesis that are markedly greater than that found in fibers that only express the adults MHC isoforms. Moreover, the data further demonstrate the existence of fiber type-dependent differences in the rates of protein synthesis.

## Discussion

This is the first study to have examined muscle fiber type-dependent changes in protein synthesis, CSA, and markers of translational capacity and efficiency, in different fiber types found within the same whole muscle. Overall, the results clearly show that perturbations such as FD and SA can induce fiber type-dependent differences in the regulation of these variables.

### Food Deprivation

It has previously been reported that 48 h of FD induces a relatively large decrease in whole skeletal muscle protein synthesis [8], [11] that is predominantly due to a decrease in translation initiation, and thus reduced translation efficiency [23], [24]. In agreement with these studies, our results at the whole muscle level show that the large FD-induced decrease in protein synthesis was associated with a similar decrease in our marker of translation efficiency, S6 Ser^240/244^ phosphorylation and relatively small changes in translational capacity as indicated by changes in total S6 protein. Previous studies have also shown that predominantly glycolytic fast-twitch whole muscles have a greater FD-induced decrease in protein synthesis than more oxidative slow-twitch whole muscles [11], [13]. In the current study, we show for the first time, the existence of fiber-type dependent reductions of protein synthesis in response to FD, with the larger glycolytic fast-twitch 2X and 2B fibers having a greater FD-induced decrease in protein synthesis than the smaller more oxidative type 1 and 2A fibers from the same whole muscle. This suggests that type 2X and 2B fibers are perhaps more sensitive to the effects of FD than the more oxidative fiber types. Furthermore, the 2X and 2B fibers also displayed the largest decreases in total S6 protein and S6 Ser^240/244^ phosphorylation, respectively. These results indicate that while the large reduction in protein synthesis in 2X and 2B fibers is predominantly due to reduced translational efficiency, the change in translational capacity also played a significant, albeit smaller, role, particularly in the case of the type 2X fibers.

The exact mechanism(s) behind the fiber type-dependent response of protein synthesis to FD remains to be determined, however, recent studies at the whole muscle level may provide some insights. For example, FD induces an increase in the expression of the atrophy-related genes, myostatin and atrogin-1, with whole fast-twitch muscles showing greater increases in expression compared with whole slow-twitch muscles [25]. Although myostatin and atrogin-1 are known to be involved in the regulation of protein degradation, they have also been implicated in the inhibition of mTOR signaling and protein synthesis, in part, by mediating the degradation of various ribosomal proteins and translation factors [26]–[28]. Thus, the greater FD-induced reduction of protein synthesis in type 2X and 2B fibers, may in part be explained by larger increases in atrogin-1 and myostatin. This is further supported by our finding that only 2X and 2B fibers experienced significant atrophy in response to FD.

Another mechanism that could help to explain the greater FD-induced decrease in type 2X and 2B fiber CSA is fiber-type dependent differences in the expression of PGC1α. Recent evidence has shown that more oxidative fiber types have higher levels of PGC1α expression than glycolytic fiber types [29]–[31], and that PGC1α may inhibit the expression of atrophy related genes [32]. Thus, lower levels of PGC1α expression in type 2X and 2B fibers [29]–[31] may leave these fiber types more susceptible to FD-induced atrophy. Finally, the smaller decrease in protein synthesis and absence of FD-induced atrophy in type 1 and 2A fibers could also be related to their more frequent use during ambulation. For example, motor units containing type 1 and 2A fibers are, in general, activated more frequently than motor units composed of type 2X and 2B fibers [33], [34]. Hence, the more frequent contractile activity in type 1 and 2A may have attenuated the FD-induced changes in protein synthesis and protein degradation and, in turn, promoted a greater resistance to changes in CSA [35].

In summary, relatively short-term FD induces fiber type-dependent changes in S6 Ser^240/244^ phosphorylation, total S6 protein, protein synthesis and fiber CSA. These fiber type-dependent responses are likely due to a combination of the intrinsic fiber type-dependent differences in the molecular mechanisms that regulate protein synthesis and degradation, as well as, fiber type-dependent use patterns. Nevertheless, further work will be needed to define the exact molecular mechanism(s) responsible for these fiber-type dependent responses.

### Fiber Type-Dependent Differences in the Basal Expression of Total Ribosomal S6 Protein and in S6 Ser^240/244^ Phosphorylation

In the process of investigating the effect of FD on translational capacity we noticed that sections from Ad Lib muscle had significant inter-fiber differences in the amount of the ribosomal S6 protein. This observation suggested that the basal expression of the ribosomal S6 protein may be fiber type-dependent. Indeed, our results showed that the total S6 protein signal intensity varied in the following order: 2B < 2X < 1 < 2A. These findings suggest that translational capacity may vary in a fiber type-dependent manner. This hypothesis is supported by the study of Habets et al [36] which showed that rat single muscle fiber 28S rRNA, a marker of ribosomal content, increased in the following order of fiber type: 2B < 2X < 2A. Moreover, our total S6 results are consistent with our previous finding that basal rates of protein synthesis varies in a similar fiber type-dependent manner (i.e. type 2B < 2X < 2A ≈ 1) [8]. Taken together, these data suggest that basal rates of protein synthesis are correlated with ribosomal content, and thus, translational capacity. We also found that the fiber type-dependent pattern for S6 Ser^240/244^ phosphorylation (2B ≈ 2X < 2A < 1) was slightly different from that observed with total S6. Specifically, the level of S6 Ser^240/244^ phosphorylation was not different between type 2X and 2B fibers. Furthermore, S6 Ser^240/244^ phosphorylation in type 1 fibers was higher than in type 2A fibers. Thus, it seems that, at least under basal conditions, rates of protein synthesis are not as closely associated with this marker of mTORC1 signaling, and presumably, translational efficiency. Nevertheless, our data clearly reveals fiber type-dependent differences in the basal levels of both total ribosomal S6 protein and S6 Ser^240/244^ phosphorylation and further work will be required to elucidate the molecular mechanisms responsible for these findings.

### Synergist Ablation

Fiber type-dependent changes in protein synthesis, CSA, S6 Ser^240/244^ phosphorylation and total S6 protein were also observed in response to SA. Specifically, SA resulted in significantly larger increases in protein synthesis in type 1 and 2A fibers compared to type 2X and 2B fibers, with the increase in type 2X fibers being intermediate to 2A and 2B fibers. Moreover, this fiber type-dependent pattern of increases in protein synthesis was largely mirrored by a similar pattern of fiber type-dependent increases in fiber CSA. For example, type 2B fibers revealed the smallest SA-induced increase in both protein synthesis and CSA, while type 2A fibers had the largest increase in both these parameters. One potential mechanism behind these observations could be the result of differences in motor unit recruitment patterns [33], [34], with the type 1 and 2A fibers being recruited, and thus mechanically overloaded, more frequently than the type 2X and 2B fibers.

Although the fiber type-dependent differences in protein synthesis and fiber CSA seem to fit well with potential differences in recruitment patterns, the SA-induced changes in total S6 protein and S6 Ser^240/244^ phosphorylation are not so congruent. For example, despite having the smallest SA-induced increase in protein synthesis, type 2B fibers had a 55–77% greater increase in total S6 protein compared to the other three fiber types. Furthermore, the SA-induced increase in S6 Ser^240/244^ phosphorylation was not different between type 2A and 2X fibers, and type 1 fibers had a larger increase in S6 Ser^240/244^ phosphorylation than type 2A fibers. These data suggest that the fiber type-specific regulation of protein synthesis and CSA in response to mechanical overload results from a complex combination of changes in translational efficiency and capacity. Interestingly, the SA-induced increase in Ser^240/244^ S6 phosphorylation, when expressed relative to the increase in total S6 protein, was 5.7-, 4.4-, 4.4- and 1.8-fold for type 1, 2A, 2X and 2B fibers, respectively. This suggests that, compared to type 1, 2A and 2X fibers, the increase in Ser^240/244^ S6 phosphorylation in type 2B fibers was largely due to an increase total S6 protein. This also suggests that, compared to type 1, 2A and 2X fibers, the increase in protein synthesis in type 2B fibers was driven to a greater extent by an increase in translational capacity than translational efficiency. Moreover, the fact that there was a relatively smaller increase in protein synthesis in type 2B fibers (Fig. 2F), despite the larger increase in total S6 protein compared to the other fiber types (∼ 2-fold), suggests that increases in translational efficiency play a more dominant role in determining the effect of SA on protein synthesis.

Apart from differences in motor unit recruitment, another possible reason for the smaller SA-induced increase in protein synthesis, CSA and Ser^240/244^ S6 phosphorylation in type 2B fibers could be related to absolute fiber size. For example, in rodent muscles, type 2B fibers are the largest of the four fiber types (Fig. 2G) and their increase in size may be limited by factors such as oxygen diffusion, or the requirement for an optimal myonuclei to cytoplasm ratio [6]. Thus, despite the SA-induced increase in total S6 protein, and presumably translational capacity, other molecular mechanisms may have acted to limit the increase in protein synthesis by restricting translational efficiency. One such mechanism could be a fiber type-dependent increase in the activation AMPK, a known inhibitor of mTORC1 signaling and protein synthesis [37]. Indeed, previous studies have shown that SA induces the activation of AMPK activity [38] and that intense exercise induces AMPK α-subunit Thr^172^ phosphorylation to a greater extent in type 2X fibers than in type 1 and 2A fibers [39]. Although the exact mechanism for enhanced AMPK activation in the faster fiber types remains to be determined, it could in part be mediated by limited oxygen diffusion and greater metabolic stress due to the lower capillary densities and blood flow in these fibers [40]. A limitation in oxygen diffusion could also induce the expression of HIF-1α and REDD1 which are also known inhibitors of mTORC1 signaling [41], [42]. In summary, our results demonstrate that SA induces fiber type-dependent increases in total S6 protein, S6 Ser^240/244^ phosphorylation, protein synthesis and fiber CSA. As with FD, the SA induced changes are likely due to complex array of factors and additional studies will be needed to define the molecular mechanisms that are responsible for these fiber type-dependent effects.

### MHC^Emb^ Positive Fibers in Synergist Ablated Muscle

Previously we have shown that SA induces an increase in the total number of muscle fibers and this effect is matched by a proportional increase in the number of fibers that express the MHC^Emb^ isoform [16]. Based on these findings we, and others, have interpreted these observations as evidence for SA-induced muscle fiber hyperplasia [16], [43]. In the current study, we again identified a population of MHC^Emb^ positive fibers in SA muscles, and using the *in vivo* SUnSET technique, we were able to measure the relative rate of protein synthesis in these fibers. Specifically, the MHC^Emb^ positive fibers in SA muscles had rates of protein synthesis that were 3.6-fold greater than that observed in sham muscle fibers. The greater rates of protein synthesis were also associated with a 2.9-fold greater amount of total S6 protein and a 6.1-fold greater amount of S6 Ser^240/244^ phosphorylation. Combined, our results suggest that SA induces new fiber formation and that these fibers possess a robust activation of mTORC1 signaling, translational efficiency, translational capacity and protein synthesis. Furthermore, we have reported that 30–40% of the total fibers in 14 day SA muscles are MHC^Emb^ positive [16], thus, these fibers are likely to account for a significant proportion of the increase in total S6 protein, S6 Ser^240/244^ phosphorylation and protein synthesis that is observed at the whole muscle level. Taken together, these results provided a clear and striking example of fiber type-dependent differences in protein synthesis that may not be evident if analyses are performed at the whole muscle level.

### Conclusion

In summary, this study has demonstrated the existence of fiber type-dependent changes in protein synthesis in fibers found within the same whole muscle. In addition, fiber type-dependent changes in CSA, total S6 protein and S6 Ser^240^/^244^ phosphorylation, were also observed in response to FD and SA. Furthermore, for the first time, we were also able to measure the relative rate of protein synthesis in putative newly formed MHC^Emb^ positive fibers that are found in SA muscles. Combined, this study highlights that the *in vivo* SUnSET methodology can be used for investigating fiber type-specific responses in protein synthesis to a variety of physiological stressors, or to potential therapies designed to treat various skeletal muscle pathologies. Moreover, our findings illustrate that changes found at the whole muscle level may not accurately reflect the changes that occur within the individual muscle fiber types.

## Supporting Information

Figure S1 **Food Deprivation Induces Fiber Type-Dependent Changes in Protein Synthesis and Cross-Sectional Area.** Plantaris muscles obtained from control (Ad Lib) and 48 h food deprived (FD) mice were frozen adjacent to one another, cross-sectioned, and then subjected to immunohistochemistry for rates of protein synthesis (puromycin) and different fiber types as described in Figure 1. (**A–H**) Frequency histograms representing the effect of FD on the relative rate of protein synthesis (i.e. puromycin staining intensity) (**A, C, E, G**), and cross-sectional area (**B, D, F, H**), within a given fiber type. Inset values are presented as the mean ± SEM (n  = 60–250 fibers / group from 5 independent pairs of muscles). * Significant effect of FD, (P<0.05).
(TIF)

Figure S2 **Synergist Ablation Induces Fiber Type-Dependent Changes in Protein Synthesis and Cross-Sectional Area.** Plantaris muscles obtained from control (Sham) and 10 d synergist ablated (SA) mice were frozen adjacent to one another, cross-sectioned, and then subjected to immunohistochemistry for rates of protein synthesis (puromycin) and different fiber types as described in Figure 2. (**A–H**) Frequency histograms representing the effect of SA on the relative rate of protein synthesis (i.e. puromycin staining intensity) (**A, C, E, G**), and cross-sectional area (**B, D, F, H**), within a given fiber type. Inset values are presented as the mean ± SEM (n  = 84–500 fibers / group from 6 independent pairs of muscles). * Significant effect of SA, (P<0.05).
(TIF)

Figure S3 **Fiber Type-Dependent Differences in Basal Ser^240/244^ Phosphorylated and Total Ribosomal S6 Protein.** Muscle sections from Ad Lib mice were subjected to immunohistochemistry for different fiber types and Ser^240/244^ phosphorylated S6 (P-S6 Ser^240/244^) or total S6, as described in Figure 3. (**A**) P-S6 Ser^240/244^ and (**B**) total S6 in each fiber type was expressed relative to the mean value obtained in type 2A fibers from the same section. Values are means + SEM (*n*  = 59–300 fibers / group from 4–5 independent muscles). ∗ significantly different from all other fiber types, # significantly different from type 1 and 2A fibers (P>0.05).
(TIF)

Figure S4 **Food Deprivation Induces Fiber Type-Dependent Changes in Ser^240/244^ Phosphorylated and Total Ribosomal S6 Protein.** Plantaris muscles obtained from control (Ad Lib) and 48 h food deprived (FD) mice were frozen adjacent to one another, cross-sectioned, and then subjected to immunohistochemistry for different fiber types and Ser^240/244^ phosphorylated S6, or total S6, as described in Figure 3. (**A–H**) Frequency histograms representing the effect of FD on the relative staining intensity of Ser^240/244^ phosphorylated S6 (P-S6 Ser^240/244^) (**A, C, E, G**), and total S6 (**B, D, F, H**), within a given fiber type. Inset values are presented as the mean ± SEM (n  = 60–250 fibers / group from 5 independent pairs of muscles). * Significant effect of FD, (P<0.05).
(TIF)

Figure S5 **Synergist Ablation Induces Fiber Type-Dependent Changes in Ser^240/244^ Phosphorylated and Total Ribosomal S6 Protein.** Plantaris muscles obtained from control (Sham) and 10 d synergist ablated (SA) mice were frozen adjacent to one another, cross-sectioned, and then subjected to immunohistochemistry for different fiber types and Ser^240/244^ phosphorylated S6, or total S6, as described in Figure 4. (**A–H**) Frequency histograms representing the effect of SA on the relative staining intensity of Ser^240/244^ phosphorylated S6 (P-S6 Ser^240/244^) (**A, C, E, G**), and total S6 (**B, D, F, H**), within a given fiber type. Inset values are presented as the mean ± SEM (n  = 84–500 fibers / group from 6 independent pairs of muscles). * Significant effect of SA, (P<0.05).
(TIF)

Figure S6 **Cross-Sectional Area, Protein Synthesis, Ser^240/244^ Phosphorylated and Total Ribosomal S6 Protein in MHC^Emb^ Positive Fibers.** Plantaris muscles obtained from control (Sham) and 10 d synergist ablated (SA) mice were frozen adjacent to one another, cross-sectioned, and then subjected to immunohistochemistry for MHC^Emb^ and rates of protein synthesis (puromycin), Ser^240/244^ phosphorylated S6 (P-S6 Ser^240/244^), or total S6, as described in Figures 2 and 4. Frequency histograms of the (**A**) fiber cross-sectional area (CSA), (**B**) rate of protein synthesis, (**C**) amount of Ser^240/244^ phosphorylated S6 (P-S6 Ser^240/244^) and (**D**) total amount of S6 in MHC^Emb^ positive fibers of SA muscles expressed relative to randomly selected fibers from sham muscles (Mixed Sham). Inset values are presented as the mean ± SEM (n  = 152–360 fibers / group from 6 independent pairs of muscles). ∗ Significantly different from mixed sham, (P<0.05).
(TIF)
