# Supplementary figures and images for: Muscle Fiber Type-Dependent Differences in the Regulation of Protein Synthesis
Source: PLoS One. 2012 May 22;7(5):e37890. doi: 10.1371/journal.pone.0037890 (PMC3358270; doi:10.1371/journal.pone.0037890)

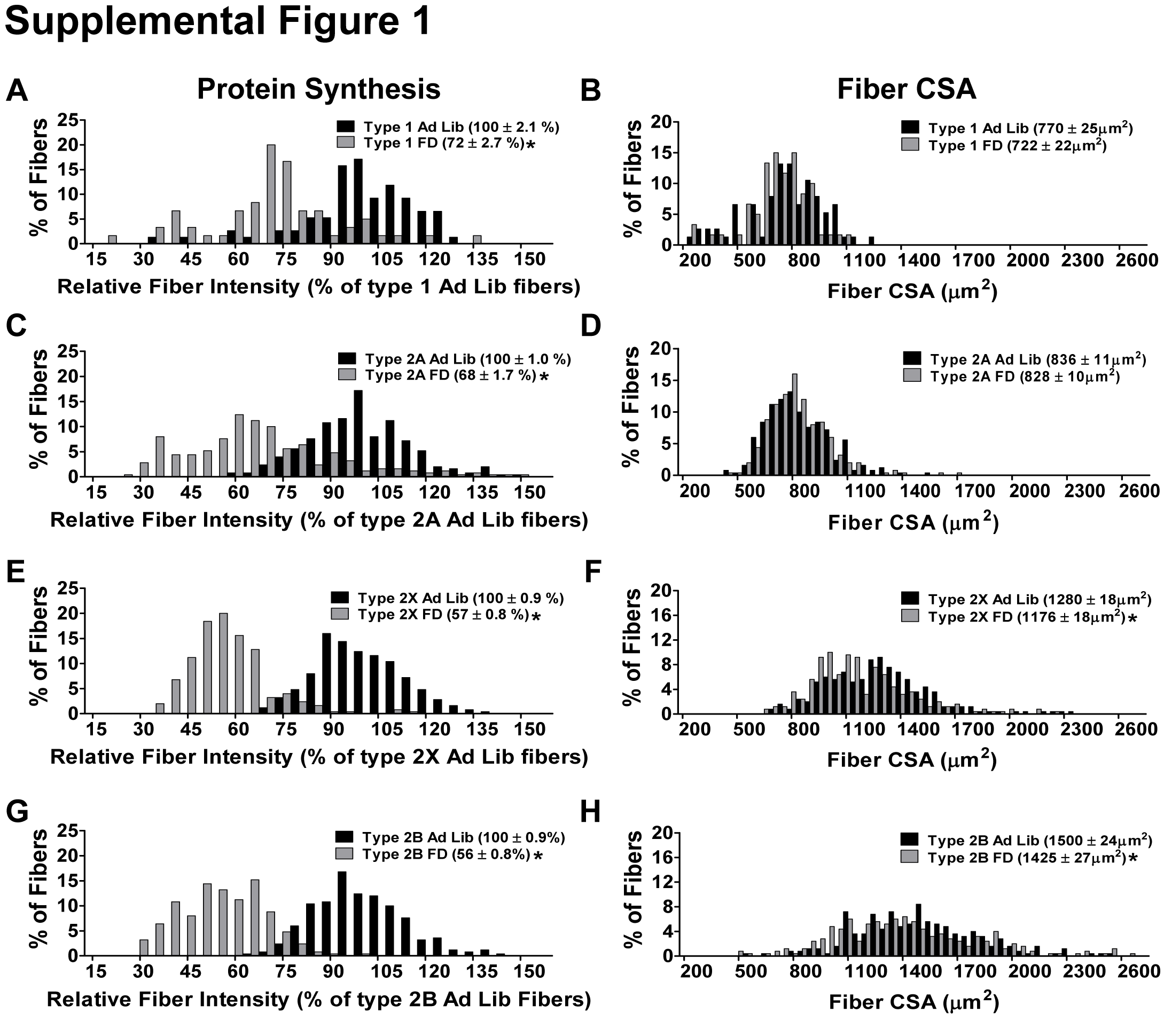

Supplement: Figure S1 — Food Deprivation Induces Fiber Type-Dependent Changes in Protein Synthesis and Cross-Sectional Area. Plantaris muscles obtained from control (Ad Lib) and 48 h food deprived (FD) mice were frozen adjacent to one another, cross-sectioned, and then subjected to immunohistochemistry for rates of protein synthesis (puromycin) and different fiber types as described in Figure 1. (A–H) Frequency histograms representing the effect of FD on the relative rate of protein synthesis (i.e. puromycin staining intensity) (A, C, E, G), and cross-sectional area (B, D, F, H), within a given fiber type. Inset values are presented as the mean ± SEM (n = 60–250 fibers / group from 5 independent pairs of muscles). * Significant effect of FD, (P<0.05). (TIF) [file pone.0037890.s001.tif]

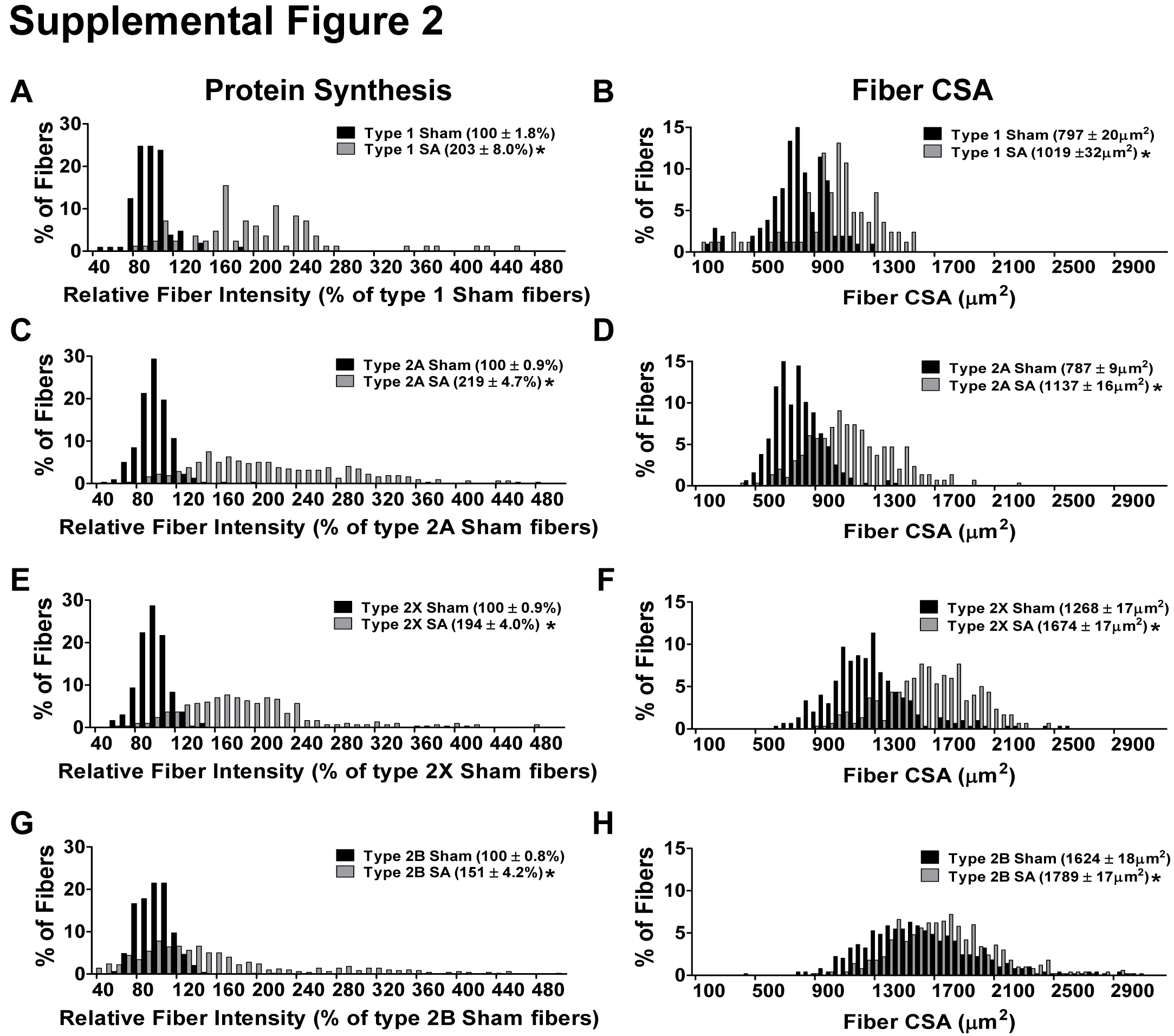

Supplement: Figure S2 — Synergist Ablation Induces Fiber Type-Dependent Changes in Protein Synthesis and Cross-Sectional Area. Plantaris muscles obtained from control (Sham) and 10 d synergist ablated (SA) mice were frozen adjacent to one another, cross-sectioned, and then subjected to immunohistochemistry for rates of protein synthesis (puromycin) and different fiber types as described in Figure 2. (A–H) Frequency histograms representing the effect of SA on the relative rate of protein synthesis (i.e. puromycin staining intensity) (A, C, E, G), and cross-sectional area (B, D, F, H), within a given fiber type. Inset values are presented as the mean ± SEM (n = 84–500 fibers / group from 6 independent pairs of muscles). * Significant effect of SA, (P<0.05). (TIF) [file pone.0037890.s002.tif]

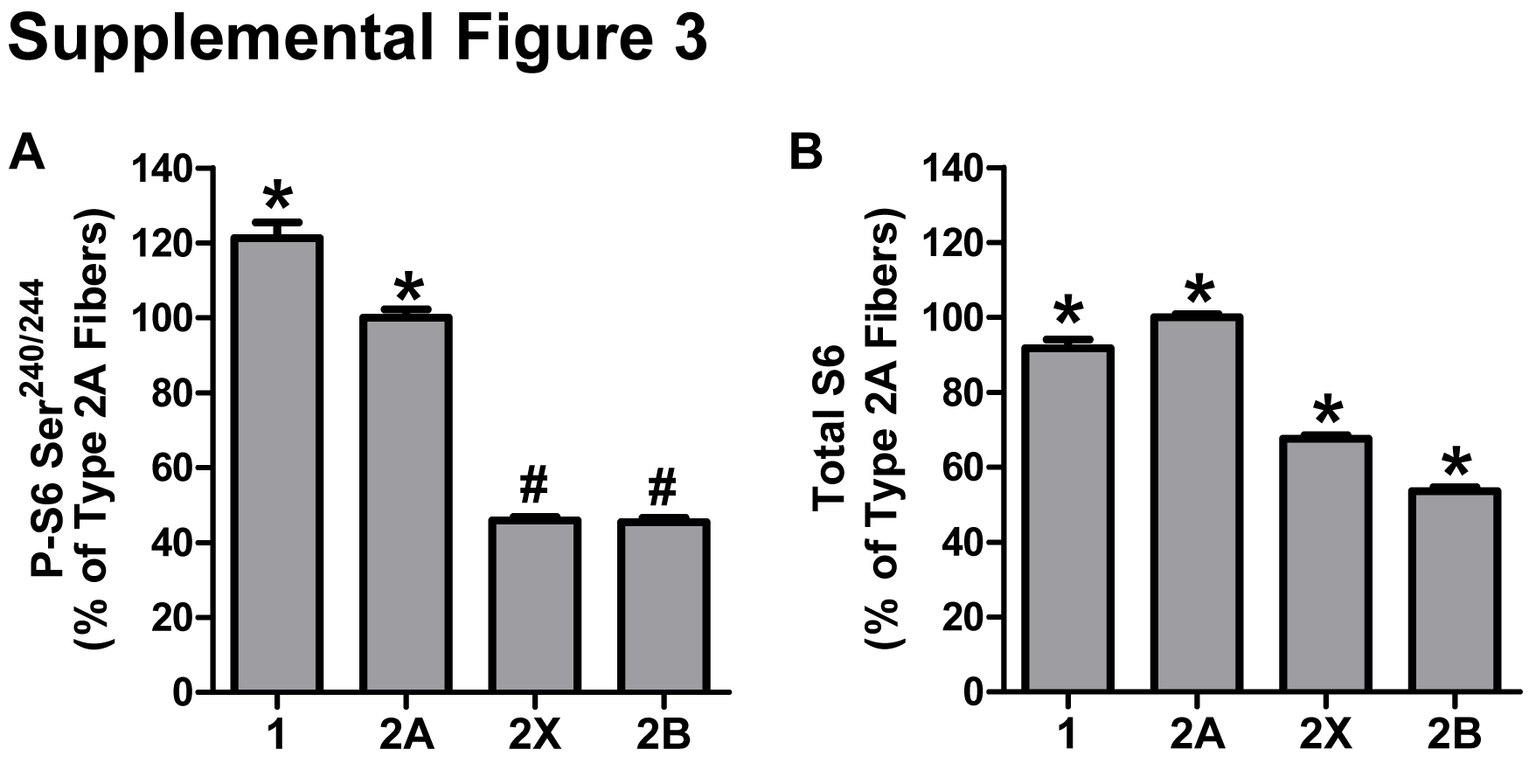

Supplement: Figure S3 — Fiber Type-Dependent Differences in Basal Ser240/244 Phosphorylated and Total Ribosomal S6 Protein. Muscle sections from Ad Lib mice were subjected to immunohistochemistry for different fiber types and Ser240/244 phosphorylated S6 (P-S6 Ser240/244) or total S6, as described in Figure 3. (A) P-S6 Ser240/244 and (B) total S6 in each fiber type was expressed relative to the mean value obtained in type 2A fibers from the same section. Values are means + SEM (n = 59–300 fibers / group from 4–5 independent muscles). ∗ significantly different from all other fiber types, # significantly different from type 1 and 2A fibers (P>0.05). (TIF) [file pone.0037890.s003.tif]

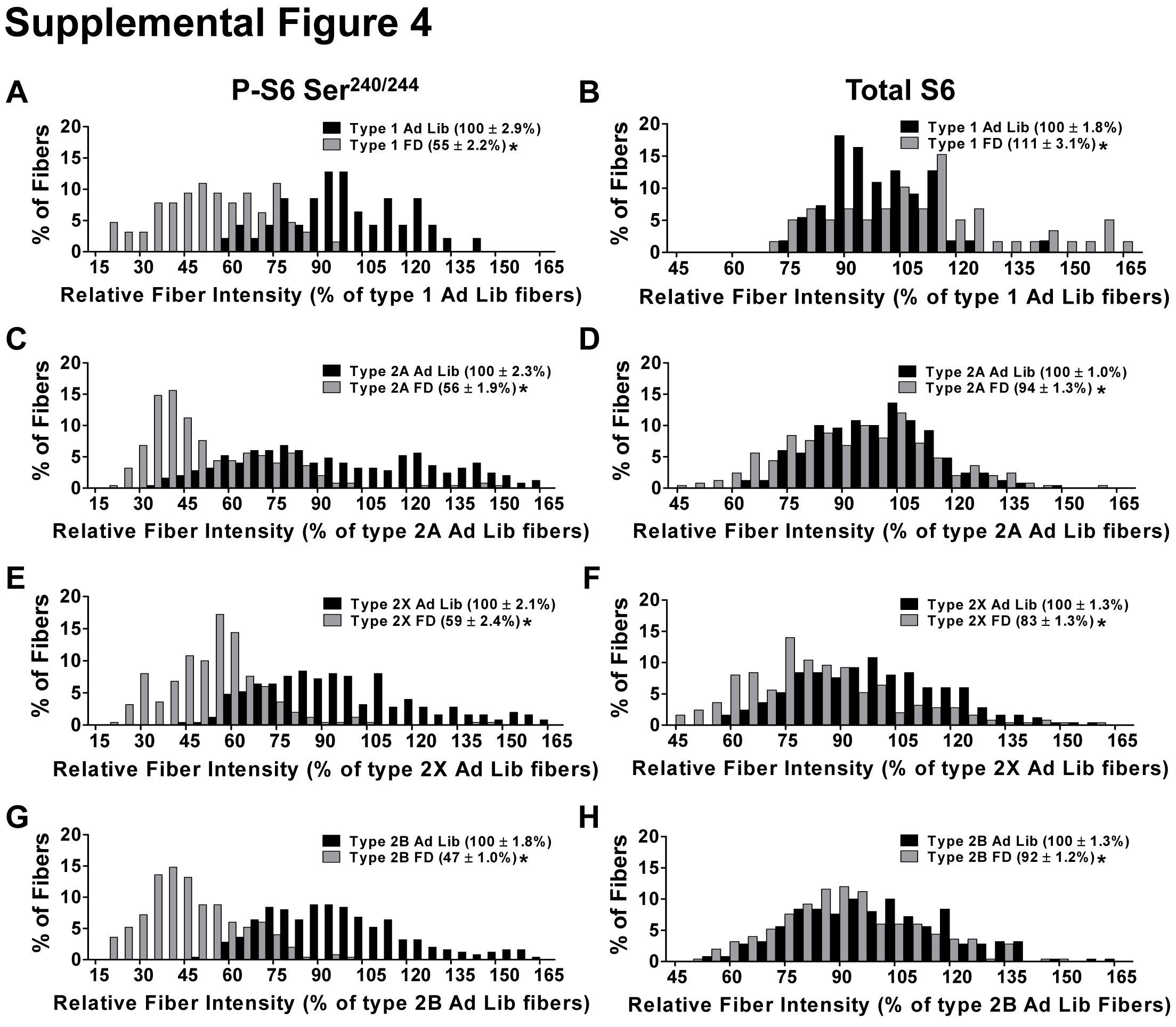

Supplement: Figure S4 — Food Deprivation Induces Fiber Type-Dependent Changes in Ser240/244 Phosphorylated and Total Ribosomal S6 Protein. Plantaris muscles obtained from control (Ad Lib) and 48 h food deprived (FD) mice were frozen adjacent to one another, cross-sectioned, and then subjected to immunohistochemistry for different fiber types and Ser240/244 phosphorylated S6, or total S6, as described in Figure 3. (A–H) Frequency histograms representing the effect of FD on the relative staining intensity of Ser240/244 phosphorylated S6 (P-S6 Ser240/244) (A, C, E, G), and total S6 (B, D, F, H), within a given fiber type. Inset values are presented as the mean ± SEM (n = 60–250 fibers / group from 5 independent pairs of muscles). * Significant effect of FD, (P<0.05). (TIF) [file pone.0037890.s004.tif]

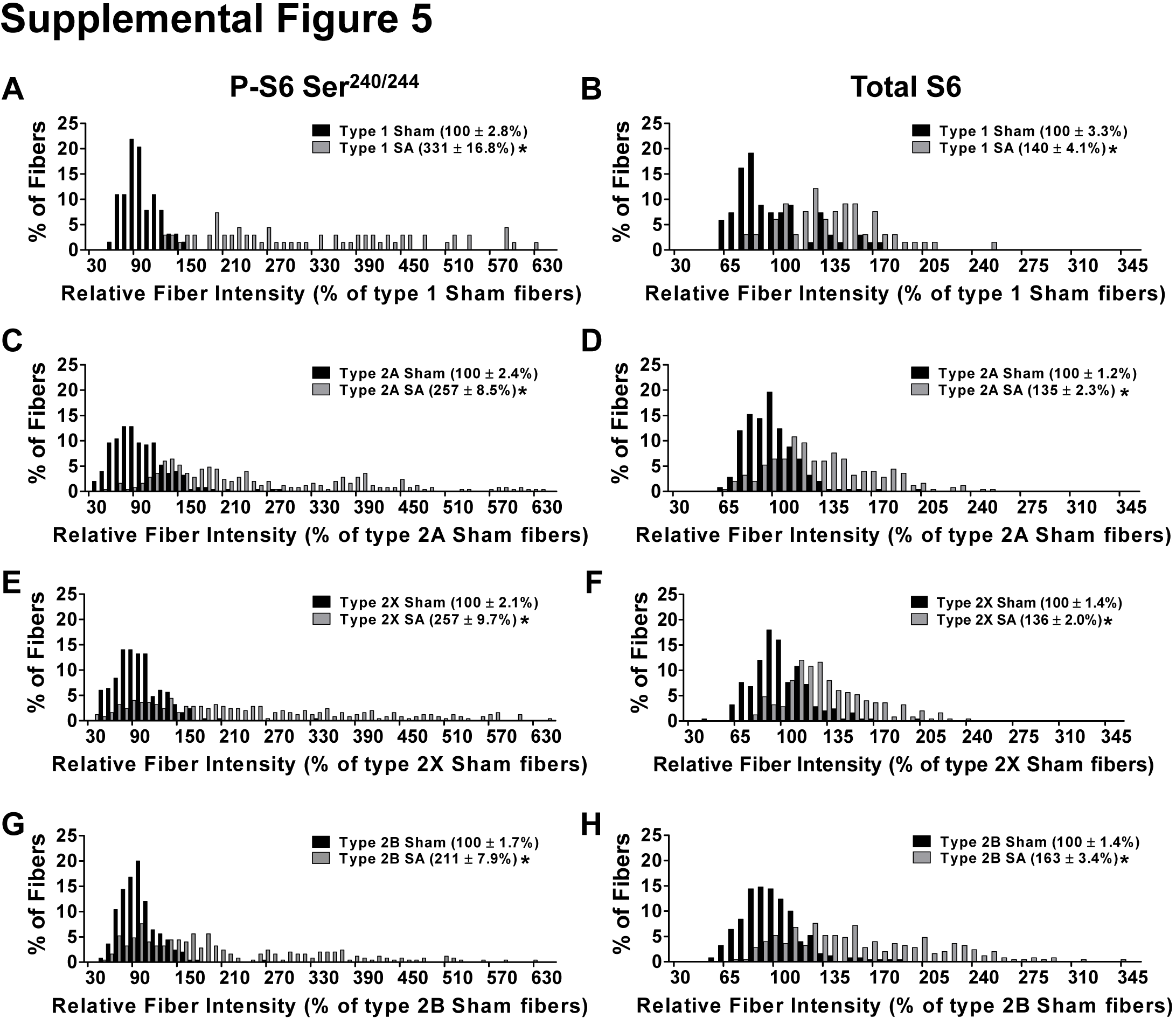

Supplement: Figure S5 — Synergist Ablation Induces Fiber Type-Dependent Changes in Ser240/244 Phosphorylated and Total Ribosomal S6 Protein. Plantaris muscles obtained from control (Sham) and 10 d synergist ablated (SA) mice were frozen adjacent to one another, cross-sectioned, and then subjected to immunohistochemistry for different fiber types and Ser240/244 phosphorylated S6, or total S6, as described in Figure 4. (A–H) Frequency histograms representing the effect of SA on the relative staining intensity of Ser240/244 phosphorylated S6 (P-S6 Ser240/244) (A, C, E, G), and total S6 (B, D, F, H), within a given fiber type. Inset values are presented as the mean ± SEM (n = 84–500 fibers / group from 6 independent pairs of muscles). * Significant effect of SA, (P<0.05). (TIF) [file pone.0037890.s005.tif]

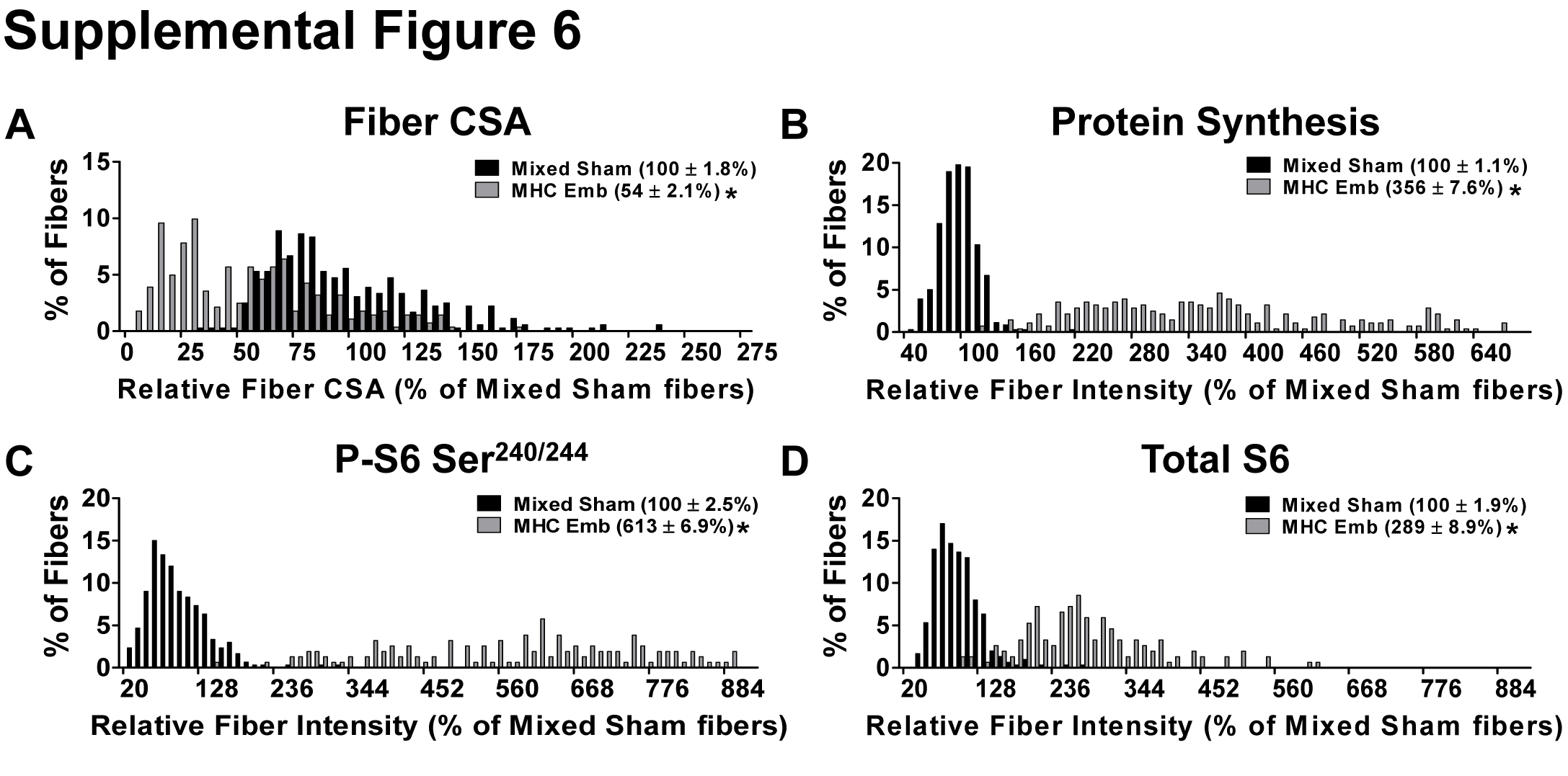

Supplement: Figure S6 — Cross-Sectional Area, Protein Synthesis, Ser240/244 Phosphorylated and Total Ribosomal S6 Protein in MHCEmb Positive Fibers. Plantaris muscles obtained from control (Sham) and 10 d synergist ablated (SA) mice were frozen adjacent to one another, cross-sectioned, and then subjected to immunohistochemistry for MHCEmb and rates of protein synthesis (puromycin), Ser240/244 phosphorylated S6 (P-S6 Ser240/244), or total S6, as described in Figures 2 and 4. Frequency histograms of the (A) fiber cross-sectional area (CSA), (B) rate of protein synthesis, (C) amount of Ser240/244 phosphorylated S6 (P-S6 Ser240/244) and (D) total amount of S6 in MHCEmb positive fibers of SA muscles expressed relative to randomly selected fibers from sham muscles (Mixed Sham). Inset values are presented as the mean ± SEM (n = 152–360 fibers / group from 6 independent pairs of muscles). ∗ Significantly different from mixed sham, (P<0.05). (TIF) [file pone.0037890.s006.tif]
